# Effects of Culture on the Balance Between Mathematics Achievement and Subjective Wellbeing

**DOI:** 10.3389/fpsyg.2022.894774

**Published:** 2022-05-26

**Authors:** Jingyi Meng, Simiao Liu

**Affiliations:** ^1^Institute on Educational Policy and Evaluation of International Students, Beijing Language and Culture University, Beijing, China; ^2^Faculty of Education, The University of Hong Kong, Pokfulam, Hong Kong SAR, China

**Keywords:** culture, mathematics achievement, subjective wellbeing, PISA, balance

## Abstract

Previous studies suggested that culture have impact on students' mathematics achievement and subjective wellbeing, but few investigated the effects of culture on the balance between them. Drawing on Hofstede's cultural dimensions theory, this study investigated the effects of culture on balance between students' mathematics achievement and subjective wellbeing. Results showed the significant effects of cultural dimensions of long-term orientation vs. short-term orientation and indulgence vs. restraint. Students from countries of high long-term orientation and low indulgence culture were more likely to get both high mathematics achievement and high SWB. Besides, wealth-related variables (family SES and GDP per capita) and gender were also found to influence the odds ratio of balance. The findings confirmed the effects of national culture on the balance between mathematics achievement and SWB. Based on the findings, this study discussed the effects of long-term orientation and restraint culture and Confucian heritage culture's potential benefit. The results indicate that mathematics educators should consider cultural differences in educational practice and stress the importance and meaning of mathematics learning.

## Introduction

Mathematics achievement and subjective wellbeing (SWB) are both critical indicators of high-performing education systems (OECD, 2019a) and play an essential role in students' lives (Steinmayr et al., 2018). As the saying goes, “all work and no play makes jack a dull boy,” implying to attach importance to balance between achievement and personal subjective wellbeing. However, previous studies revealed that the correlation between mathematics achievement and SWB was weak, which means that high-achieving students do not necessarily report higher SWB than their classmates (for review, see Suldo et al., 2006; Bucker et al., 2018). Under such circumstance, how to prepare students with both high achievement and high SWB? For educators, it is essential to learn about the antecedents of both. Although various studies have explored the determinants of students' academic achievement or SWB separately, few empirical studies investigated their common influential factors. As a result, more research is needed to explore the factors that influence the balance between achievement and SWB.

Moreover, the strives of mathematics education indisputably take place under a specific cultural environment. Consequently, it is essential to understand the potential influence of culture on both mathematics achievement and SWB. Previous comparative studies have discovered that culture plays a role in explaining disparities in mathematical achievement (e.g., Bishop, 1988; Chen and Uttal, 1988; Stevenson et al., 1993; Leung, 2006) and SWB between countries (Veenhoven et al., 1993; Inglehart and Klingemann, 2000; Diener and Biswas-Diener, 2002; Steel et al., 2018). Their conclusions, however, seem to be in conflict. For example, some behaviors valued by the high-achieving countries, such as delaying gratification and working hard, were found to reduce SWB (Oishi and Diener, 2003; Steel et al., 2018). These findings suggested that the “happiness” culture may not be compatible with the “high-achieving” culture. Consequently, it raises the issue of cultural effects on the balance between mathematics achievement and SWB.

In order to address these above issues, the present study will examine the effects of culture on the balance between students' mathematics achievement and subjective wellbeing.

## Literature Review

This section will review the related concepts and studies in order to present a picture of current research on the relationship between culture, mathematics achievement, and SWB.

### The Construct of Culture

Culture is a broad phrase that encompasses a complex set of concepts (Taras et al., 2009). In previous studies, scholars tend to define culture from different perspectives. For example, it was defined by Hofstede (1980, p. 25) as “the collective programming of the mind that distinguishes the members of one group or category of people from others.” While according to Triandis (1972, p. 4), it refers to “an individual characteristic way of perceiving the man-made part of one's environments … involves the perception of rules, norms, roles, and values.” Moreover, many definitions continue to evolve over time (for reviews, see Taras et al., 2009; Steel et al., 2018).

Among dozens of definitive models of culture, most of them share several common elements. First, it is generally agreed that culture refers to relatively stable values shared by a group or society for a long period (Taras et al., 2009). Second, culture is viewed as a multilevel construct. Many scholars use the “onion” metaphor to separate culture for different layers (Hofstede, 1980; Trompenaars, 1993). According to Hofstede (2001), the different layers of the culture “onion” are values, rituals, heroes, and symbols. The values lie at the center of the “onion,” symbols represent the outer layers, and rituals and heroes form the middle layers. Third, culture is multidimensional. Almost all the existing models comprise various dimensions of values and attitudes (Taras et al., 2009).

In the research field of mathematics education and subjective wellbeing (e.g., Hu et al., 2018; Steel et al., 2018), Hofstede (1980) cultural dimensions theory is the most popular one which combines the aforementioned common features. Hofstede (1980) original model divided culture into four dimensions, including power distance, individualism-collectivism, masculinity-femininity, and uncertainty avoidance. (1) *Power distance (PDI)* refers to the extent to which members of organizations or countries believe that power is equally distributed. A higher PDI level indicates a well-established societal hierarchy, whereas a lower one shows an equitably distributed power structure. (2) *individualism-collectivism (IDV)* indicates the extent to which people are integrated into groups. (3) *masculinity-femininity (MAS)* is a sociocultural trait indicating the division of emotional roles between genders. Higher MAS reveals a society with a preference of achievement, assertiveness, and heroism, while lower MAS reveals a “feminine” society that prefers cooperation, modesty, and caring for the weak. (4) *Uncertainty avoidance (UAI)* is a term that describes people's aversion toward uncertainty. People from higher UAI countries treat uncertainty as threats, while those from low UAI countries would be more accustomed to unfamiliar situations.

In addition, Hofstede further proposed the other two dimensions in the following research (Hofstede et al., 2010). That is (5), *Long-term orientation vs. short-term orientation (LTO)*. This dimension is halfway between long-term and short-term perspectives. The long-term pole emphasizes the importance of virtues that lead to future rewards, such as perseverance and thrift, whereas the short-term pole emphasizes past and present virtues, such as reverence for tradition and maintaining one's “face.” (6) *Indulgence vs. restraint (IND)*. This dimension refers to the degree of freedom that society allows for human desires. A high IND civilization allows for relatively unrestricted gratification of fulfilling human desires, whereas a low IND society regulates the gratification of needs.

Hofstede's framework is widely accepted by cross-cultural scholars. In the review of Taras et al. (2009), more than 120 existing survey instruments of culture shared one or more common dimensions with Hofstede's approach. The finding reveals that the framework is well-representative of culture when it comes to defining and measuring it. Therefore, the present study will use Hofstede's six cultural dimensions to describe culture. Furthermore, in line with the traditional way in mathematics education (e.g., Stankov, 2010; Leung, 2014), the culture used in this study refers to the cultural value, which belongs to the core module of the “onion” construct of culture.

### The Influence of Culture on Mathematics Achievement

International large-scale surveys (e.g., TIMSS and PISA) always reported substantial national differences in mathematics achievement. Such across-national differences have been the focus of mathematics education for a long time (e.g., Hess et al., 1987; Bishop, 1988; McInerney et al., 1997). Among various factors that contribute to the achievement differences, culture is a non-negligible one. For example, when comparing the differences between East-Asian and Western countries, scholars discovered that cultural factors may be more effective than economic determinants in explaining the better performance of East-Asian students (Chen and Uttal, 1988; Stevenson et al., 1993; Leung, 2006).

The effects of culture could take place at the national level. In the study of Hu et al. (2018), the cultural dimension of long-term orientation was significantly related to mathematics achievement. Specifically, countries with a higher level of long-term orientation would outperform in mathematics achievement. Besides, many studies explored the cultural difference in the national curriculum (Leung's, 1992; Li and Ginsburg, 2006). For instance, in Leung's (1992) comparative study, he found that the U.K. curriculum stressed the intrinsic aims, whilst the Chinese curriculum did not, and such a difference could be explained by the cultural differences between individualistic and collectivistic orientations. This explanation is also in line with Hofstede's dimension of individualism-collectivism. In addition, Li and Ginsburg (2006) showed that, compared with the U.S., the East-Asian countries' textbook showed a higher level of classification, but their teachers had a lower level of autonomy in selecting ways for knowledge presentations. The differences were explained by the cultural values of authority relations, which align with the cultural dimension of power distance.

The effects could also take place at the classroom and individual levels. Based on observation of classroom teaching in different cultures, Leung (2001) summarized six differences between the Eastern and Western mathematics educations, such as rote vs. meaningful learning and extrinsic vs. intrinsic motivation, and further argued that the characteristics in East-Asian classrooms could attribute to the Confucian heritage culture. In terms of the individual-level effects, Hu (2019) found that student-espoused cultural values, such as dimensions of individualism-collectivism and power distance, were significantly associated with their mathematics achievement in some cultural groups of China.

In conclusion, culture has gotten a lot of attention in mathematics education, and it's been found to be an important element in explaining pupils' math achievement. However, the effects of culture on mathematics achievement may not be consistent with that on students' subjective wellbeing. As mentioned above, high-achieving students do not necessarily report higher SWB than low-achieving ones (Suldo et al., 2006; Bucker et al., 2018). Moreover, some cultural dimensions contributing to high achievement may sacrifice students' SWB. For instance, Leung (2014) found that East-Asian students had negative attitudes toward mathematics, and the culture could be a possible explanation. Therefore, the influence of culture on SWB will be reviewed in the next section to extend the understanding beyond achievement.

### The Influence of Culture on Subjective Wellbeing

Similar to mathematics achievement, previous international surveys also reported consistent differences in SWB across countries (Veenhoven et al., 1993; Inglehart and Klingemann, 2000). The differences could partially attribute to the wealth and related predictors, but not all (Diener et al., 2003). Furthermore, the effects of wealth-related factors on SWB normally decrease as national economic situation improve (Diener and Biswas-Diener, 2002). It was assumed that the wealth-related variables matter most to SWB before meeting basic human needs (Inglehart and Klingemann, 2000). In this way, scholars turned to look for explanatory factors other than wealth. One interesting finding was that the cultural differences seemed to parallel to the SWB differences. For example, European Americans always reported higher life satisfaction and less unhappiness than Asian Americans, though living in the same country (Okazaki, 2000). One explanation of this phenomenon is the cultural differences in self-evaluations and attributions (Diener et al., 2003). It has been found that North Americans tended to judge themselves with self-enhancement, while East Asians tended to do this with self-criticism (Oishi and Diener, 2003). This kind of self-evaluation differences (self-enhancement or self-criticism) was related to the cultural focus on individualism and collectivism.

Another cultural difference exists in people's strategies for tradeoffs. The most common thing would be the tradeoff between immediate happiness and future goals. A previous study suggested that Asian-American students would feel better when pursuing future goals, while Caucasian students preferred immediate hedonic activities (Asakawa and Csikszentmihalyi, 2000). Similar things were also found between European and Asian Americans (Oishi and Diener, 2003). Asian American students were very perseverant in reaching the goal of a particular task, but European Americans would be more likely to give up and switch to other tasks if they cannot do well in a certain task. As a consequence, the switching strategy of European American students would lead to more enjoyment. These findings revealed the difference in cultural dimensions of long-term orientation and indulgence. It seems that culture in favor of immediate happiness would lead to higher enjoyment. However, this tradeoff method may not be beneficial to long-term goals like learning (Diener et al., 2003).

Furthermore, it seems that the “happy” culture may not be consistent with the “high-achieving” culture. In the meta-analysis of Steel et al. (2018), it was found that happy nations had specific features of low power distance, low uncertainty avoidance, high femininity, and high individualism. However, those features seem to contradict East-Asian Culture, which is always featured by high mathematics performance, with relatively high collectivism, high uncertainty avoidance, and high power distance. Moreover, some behaviors valued by Confucian heritage culture, such as delaying gratification and working hard, were found to reduce SWB (Oishi and Diener, 2003; Steel et al., 2018). The findings were also in line with the reports of large-scale assessments, which suggested that East Asian students had negative motivation but high mathematics performance (Mullis et al., 2016).

### The Present Study

The different patterns between culture-achievement and culture-SWB relationships may challenge mathematics educators on preparing students with proficient mathematics literacy and abundant SWB. As discussed above, the efforts of mathematics education indisputably take place under a specific cultural environment. Therefore, it is essential to understand the effects of culture on balance between students' mathematics achievement and SWB. Although previous studies explored the effects of culture on mathematics achievement and SWB separately, few focused on the balance. Therefore, this study attempted to bridge the gap by exploring the effects of national culture on the balance between high mathematics achievement and high SWB.

## Methods

### Data Source and Sample

In order to operationalize culture for study, every research that attempts to investigate culture-related phenomena of any kind must first define and categorize it correctly. This is a difficult task because culture is a complex and dynamic entity. We used Hofstede's more current six-dimensional model of culture in building our conceptual framework. Despite the fact that Hofstede's culture model is the most generally used and influential model of a nation-centered cultural framework (Steenkamp, 2001; Dwyer et al., 2005), it has been criticized. McSweeney (2002), for example, critiques Hofstede's conceptualization of national culture and points out that culture transcends political borders. As a result, nation states are not an appropriate level of study. Besides, giving an overly simplified dimensional definition of culture and neglecting the presence of cultural variety within countries seems inappropriate (Minkov and Hofstede, 2014).

Despite these concerns, Hofstede's approach has influenced a large number of research that use one or more of the dimensions to explain observed disparities between nationalities (Botero and Van Dyne, 2009; Landau, 2009; Kaasa, 2015). It is still widely regarded as a well-founded method for describing culture in the sense employed in this study (Sivakumar and Nakata, 2001; Secter, 2003; Venaik and Brewer, 2008). Hofstede's framework of national culture is considered to be a valid and useful instrument in quantifying national culture in a relatively large number of countries (Gibson et al., 2006). The data was collected from more than 116,000 respondents in over 50 countries and regions. Subsequent studies validating the earlier results have included respondents such as commercial airline pilots, students, civil service managers, “up-market” consumers and “elites.”

It is worth noting that country-level research, particularly which based on Hofstede's national scores, has been chastised for committing an ecological fallacy (McSweeney, 2002; Sharma, 2009; Henrich et al., 2010). We agree with this criticism, noting that a person's cultural values cannot be defined solely by his or her nationality. Nonetheless, cross-country variances in mean values, as well as considerable intra-country differences, cannot be denied. For example, Huang et al. (2019) founded that the university teachers from China and Spain showed difference on their intentions to use technology, due to their perceived cultural preferences were quite different. Our research takes a multi-level approach. We anticipate that a country-level analysis will provide macrolevel insights into behavior differences between countries, complementing the findings of an individual-level study. Therefore, these criticisms do not undermine the fundamental premise of the current study: culture have impact on students' mathematics achievement, subjective wellbeing, and the balance between them.

The data source consists of records from three databases—PISA 2018 database (OECD, 2019b), Hofstede's cultural database (Hofstede, 2015), and the International Monetary Fund database (IMF, 2018). First, the PISA 2018 database provides information about 15-year-old students' mathematics achievement and family background variables (e.g., family SES) in 79 participating countries/economies. Second, the present study included the GDP per capita records in 2018 from the IMF database to represent each participating country's national economic development. Third, Hofstede's cultural database contains a series of data collected between 1971 and 2015, representing the national culture value from six dimensions. Although the timeslot of the cultural database is different from PISA 2018, it is reasonable to link these databases at the national level (e.g., Chiu and Klassen, 2010; Hu et al., 2018), since the national culture remains very stable for an extended period (Hofstede et al., 2010).

The intersection of PISA 2018 and Hofstede's cultural databases includes 56 countries/economies, in which 10 ones did not participate in the wellbeing survey of PISA 2018. After excluding the 10 countries, the remaining dataset contains 355,042 students from 46 countries/economies. Table 1 demonstrates the descriptive statistics of interested variables for these countries/economies. The missing data of SES (2.3%) and SWB variables (Positive feeling, 8.7%; Life satisfaction, 6.8%; Meaning in life, 8.3%) were imputed with the EM algorithm by SPSS 20.0 (Graham, 2009) (See Appendix for details).

**Table 1:** Descriptive statistics of variables.

**Variables**	**Mean**	**SD**	**Description**
*Student-level*			
Mathematics achievement	470.36	97.51	Student mathematics performance. Min = 5.22, max = 915.10
Life satisfaction	7.09	2.49	The first dimension of subjective wellbeing. It represents one's reflective assessment of general life satisfaction. Min = 0, max = 10
Positive feeling	0.09	0.96	The second dimension of subjective wellbeing. It represents one's positive feelings of happiness, joyfulness, and cheerful. Min = −3.07, max = 1.24
Meaning in life	0.10	0.94	The third dimension of subjective wellbeing. It represents one's sense of meaning and purpose in life. Min = −2.15, max = 1.74
Gender			Girl = 49.97% boy = 50.03%
SES	−0.31	1.11	PISA index of economic, social and cultural status. Min = −7.75, max = 3.96
*Country-level*			
Log GDP per capita	10.29	0.52	Min = 8.98, max = 11.46
Power distance	60.28	20.89	Min = 11, max = 104
Individualism	45.13	21.68	Min = 13, max = 91
Masculinity	49.20	21.44	Min = 5, max = 110
Uncertainty avoidance	71.07	21.74	Min = 29, max = 112
Long-term orientation	53.28	22.43	Min = 13, max = 100
Indulgence	45.48	20.05	Min = 13, max = 97

### Variables

*The dependent variable* is the balance between mathematics achievement and subjective wellbeing. This study defined “balance” as a dichotomous variable, with “1” representing a state of getting both high mathematics achievement and high SWB, and “0” for otherwise. The mathematics achievement and three SWB variables (life satisfaction, positive feeling, and meaning in life) were retrieved from PISA 2018 databases. First, this study classified students into three categories according to their mathematics achievement. That is, high achievement (ranking 0–25%), medium achievement (25–75%), and low achievement (75–100%). Second, the same approach was applied to classified students according to their scores in three dimensions of subjective wellbeing, such as high life satisfaction or medium positive feeling. Students were then classified as high SWB for at least two high scores and one medium score out of three SWB dimensions. Third, the variable balance was assigned to 1 when a student got both high mathematics achievement and high SWB; otherwise, 0.

*Independent variables*. The independent variables were the six cultural dimensions in Hofstede's cultural databases, including power distance, individualism, masculinity, uncertainty avoidance, long-term orientation, and indulgence.

*Control variables*. At the national level, this study included the log GDP per capita as the control variable. At the student level, the control variables were gender and family SES (PISA index of economic, social, and cultural status).

The descriptive statistics of all the variables mentioned above were demonstrated in Table 1.

### Analysis

Because students were nested in each country/economy, such a clustered structure requires applying the multilevel linear model (Cohen, 1988; Raudenbush and Bryk, 2002). A two-level logistic regression model was established to examine the influence of culture on students' balance between mathematics achievement and subjective wellbeing. In this model, the dependent variable is the balance between mathematics achievement and subjective wellbeing. As aforementioned in the variable section, this variable was assigned to 1 when a student got high mathematics achievement and high SWB; otherwise, 0. That is based on the following considerations:

Firstly, we used the upper Quartile to represent the advantaged students. According to PISA 2018 Technical Report, the students in top quarter are considered as the advantaged. For example, PISA 2018 results point out that socio-economically advantaged students are students in the top quarter of the PISA index of economic, social and cultural status (ESCS) in their country/economy, socio-economically disadvantaged students are students in the bottom quarter of the PISA index of economic, social and cultural status (ESCS) in their country/economy.

Besides, students are classified as high SWB for at least two high scores and one medium score out of three SWB dimensions. The procedure followed methods used by relevant research. For example, Huppert and So (2013) proposed a categorical diagnosis for flourishing that required a strong endorsement of positive emotion, plus a strong endorsement of four out of five “positive characteristic” features and three out of four “positive functioning” feature. It should be noted that selecting thresholds according to data distribution makes Huppert and So's model the only one in which individual flourishing depends on how well others are doing (Hone et al., 2014).

The independent variables were six cultural dimensions, and the control variables were log GDP per capita, gender, and family SES. The multilevel model was conducted by HLM 6.0. Before establishing the model, continuous variables were standardized into *Z* scores, and the student weight (SENWT) provided by PISA 2018 was used to estimate statistics.

## Results

### Partitioning Variation in Student Balance Between Mathematics Achievement and SWB

Due to the logistic distribution feature, the level-1 unexplained variation is always equal to π^2^/3, which is 3.29 (Goldstein et al., 2002). The results of the null model showed that the variation in level-2 is 0.883 (see Table 2). Therefore, the ICC is 0.833/(0.833 + 3.29) = 0.202. According to Cohen (1988), an ICC >0.059 indicates a non-negligible within-cluster dependence. Thus, it is necessary to apply a multilevel model.

**Table 2:** Two-level logistic regression models for balance between mathematics achievement and subjective wellbeing.

	**Null model**		**Control model**		**Full model**	
	**Est**.	**S.E**.	**Est**.	**S.E**.	**Est**.	**S.E**.
**Fix effect**						
*Student level*						
Gender			0.434^***^	0.024	0.434^***^	0.024
Family SES			0.818^***^	0.049	0.819^***^	0.049
*Country level*						
Ln GDP per capita			0.710^***^	0.156	0.562^**^	0.167
Power distance					−0.043	0.090
Individualism					0.174	0.092
Masculinity					−0.083	0.067
Uncertainty avoidance					0.131	0.111
Long-term orientation					0.323^**^	0.094
Indulgence					−0.212^**^	0.071
**Random effect**	**Variance**		**Variance**		**Variance**	
Country level	0.883		0.538		0.335	
Student level	3.29		3.29		3.29	

** *p < 0.01;*

*** *p < 0.001*.

### The Influence of Culture on Balance Between Mathematics Achievement and SWB

The two-level logistic regression model results could indicate which factors would enhance students' probability of reaching a state of balance, that is, getting both high mathematics achievement and high SWB. The control model showed that gender, family SES and GDP per capita were all the significant factors contributing to students' balance. For example, the standardized coefficient of gender (0 = female, 1 = male) is 0.434, suggesting that boys were more likely to reach a balanced state than girls. In more detail, the odds ratio between boys and girls was 1.543 (*e*^0^.434). Besides, both family SES and Ln GDP per capita could also enhance the odds of being balanced.

After including cultural variables in the full model, the significance level of Ln GDP per capita declined slightly. In addition, it can be seen that two out of six cultural dimensions were significantly related to the log odds of the balance. Specifically, the dimension of long-term orientation had a positive effect on the state of balance. The odds of the state of balance would increase by 1.38 (*e*^0.323^) times for the same student if s/he lived in another country with a one-standard-deviation-higher score on the long-term orientation dimension. Conversely, the indulgence dimension coefficient was negative, indicating that the odds would drop to about four-fifth (0.81, *e*^−0.212^) if the scores of indulgences increase by one standard deviation.

## Discussion

### The Effects of Long-Term Orientation and Restraint Culture

In this study, national culture was found to have strong associations with the balance between mathematics achievement and SWB. Specifically, the cultural dimensions of long-term orientation and restraint were significantly related to better balance.

The result is in line with previous studies on mathematics education. First, in terms of the long-term orientation, comparative studies between East-Asian and Western countries suggested that some behaviors in line with the long-term orientation culture, such as delaying gratification and working hard, may contribute to the high performance of East-Asian students (Li, 2002; Leung, 2014). For example, students from long-term culture societies emphasize the importance of education and believe deeply in their own efforts (Leung, 2014). Moreover, long-term culture could promote people's self-regulation ability and delayed gratification, which are essential for successful learning (Bembenutty and Karabenick, 2013). Second, according to Hofstede's cultural dimensions theory, the restraint culture is the opposite pole to indulgence. A high indulgence society allows relatively free gratification of fulfilling human desires, while high restraint indicates a society of restraint that controls the gratification of needs. Therefore, the restraint culture mechanism may overlap with that of long-term orientation in terms of delaying gratification. It is also interesting to notice that restraint culture was not found to be related to mathematics achievement in previous studies (Hu et al., 2018; Hu, 2019). In this way, the restraint culture may be influential when considering mathematics achievement and SWB simultaneously. That is, the long-term culture could contribute to the value of virtues oriented toward future rewards, but it may need the complement of restraint culture in resisting the short-term desires.

However, the findings revealed some differences with research on SWB. Some previous studies found that culture favoring immediate happiness would lead to higher enjoyment (Diener et al., 2003; Oishi and Diener, 2003), but our study supported the benefits of long-term orientation and restraint cultures. The contradictory results could attribute to the tradeoff between immediate happiness and future goals. It has been suggested that values in favor of immediate happiness may not benefit long-term goals, such as mathematics learning (Diener et al., 2003). According to Maslow's needs theory, subjective wellbeing would be determined by different levels of need-satisfaction. Besides, compared with pursuing lower-level hedonic pleasant, pursuing a meaningful life would produce more desirable results, such as more profound happiness, serenity, and richness of inner life (Maslow, 1981). The goal of pursuing a meaningful life is congruent with the values of long-term orientation and restraint culture (Hofstede et al., 2010). The orientation to pleasure is quite common and easy, but the pursuit of meaning requires more effort and control. To this end, our findings supported the benefit of long-term orientation and restraint culture on the quality of SWB—high SWB along with high mathematics achievement.

### The Potential Benefit of Confucian Heritage Culture

Previous studies showed the challenge of keeping a balance between high achievement and high SWB (Suldo et al., 2006; Bucker et al., 2018). The complicated relationship between mathematics achievement and SWB raises concerns on whether there is a tradeoff between mathematics achievement and SWB. On the one hand, it is obvious that the benefits of mathematics learning are not always instantaneous and rely heavily on cumulative effects over time. What is more, the process of learning mathematics is full of obstacles and frustrations (Schoenfeld, 1985; Op't Eynde et al., 2007), which may reduce students' SWB. On the other side, the strategies for immediate happiness may not benefit long-term learning goals (Diener et al., 2003). Therefore, it seems that there is a tradeoff. This study found that both mathematics achievement and SWB were related to the national culture. In more details, students would be more likely to get both high mathematics achievement and high SWB in a society with high long-term orientation and low indulgence culture.

It is worth noting that the two influential cultural dimensions were not included in Hofstede's original model. As a consequence of his subsequent studies in East-Asian countries, Hofstede added the dimensions of long-term orientation vs. short-term orientation and indulgence vs. restraint into his framework (see in Hofstede et al., 2010). To some extent, this interesting finding may indicate the unique value of East-Asian culture, especially the value of Confucian heritage culture. Unlike rational utilitarianism thoughts on the quality of life, Confucian heritage culture stresses more on life's meaning (Sundararajan, 2005). Under the Confucian philosophy, people tend to answer “what is a good life” by a strong evaluation of the meaning (Taylor, 1985). The underlying belief of their evaluation is a moral map. A good example is a Confucian philosopher Mencius's proverb (Legge, 1861), “*fish are my favorite; bear's paws are also my favorites. If I cannot have both, I will choose bear's paws over fish*,” in which the fish and bear's paws were metaphors of life and righteousness. The emphasis on the meaning of life could provide the horizon for people's evaluation of life quality. In this way, the goal of learning could be consistent with the pursuit of a good life for Confucian heritage culture learners. Besides, different from the happy culture emphasizing on the high emotional arousal, Confucian heritage culture seeks for the balance state between extreme positivity and negativity, such as serenity, inner harmony, and mindfulness (Averill and More, 2000; Kitayama and Markus, 2000). The emphasis on balance may contribute to a better balance between high mathematics and high SWB.

The above discussion is not to demonstrate any exclusive advantage of Confucian heritage culture, but to search for possible explanations from Confucian heritage culture. Moreover, this study's finding was within the 46 participating countries of PISA 2018, which was not limited to Confucian heritage culture countries. Although this study's findings were in line with some Confucian heritage values, it should be noted that those values of long-term orientation and restraint could also be found in other societies, for instance, in Muslim and East European countries. In this way, shared values could be explained through the lens of different cultural perspectives. Therefore, it is more important to establish the indigenous identities for mathematics education based on deep-rooted cultural values (Leung, 2001), which is still the weakness for East Asian countries. The above discussion provides the possibility of establishing the identities of Confucian heritage culture in mathematics education.

### The Implications for Mathematics Education

The balance between mathematics achievement and SWB is essential for students, education systems, and societies. Proficient mathematics skills and affluent SWB were found to be STEM leaders' characteristics and contribute a lot to economic development (McCabe et al., 2020). This study explored the effects of national culture and other variables on the balance between mathematics achievement and SWB, which could provide some implications.

First, mathematics educators should stress the importance of education and encourage students to pursue the long-term goal of mathematics learning. As suggested by previous studies, students need to be perseverant in mathematics learning (Schoenfeld, 1985; Op't Eynde et al., 2007), but the process may reduce their SWB (Oishi and Diener, 2003; Steel et al., 2018). This study showed the positive effects of long-term orientation and restraint culture. From the Confucian heritage culture, the positive effects may derive from the emphasis on education and meaning. Specifically, working hard in mathematics learning would be more meaningful if students believe in the value of education, and success in learning could in turn bring them great satisfaction. Therefore, it is important to help students realize the value and meaning of mathematics learning. A variety of studies have investigated the factors that influence students' attitudes toward mathematics learning, such as school-related factors (e.g., teaching materials, classroom management, teacher knowledge, guidance, beliefs) and family-related factors (e.g., educational background, parental expectations) (Cheung, 1988; Mata et al., 2012; Tan, 2017). A common feature of those factors is to help students to find the meaning in mathematics learning. However, there is a widespread problem that mathematics instruction emphasizes too much on developing procedural knowledge, with limited attention to conceptual knowledge (Rittle-Johnson, 2019). Many students do not develop sufficient conceptual knowledge and fail to realize the connections of concepts and relations with their daily lives (Kilpatrick et al., 2001; Rittle-Johnson, 2019). In this aspect, mathematics educators should confirm the importance of developing both types of knowledge and impart the underlying meaning of mathematics knowledge.

Second, mathematics educators should search for the practice and establish theory compatible with indigenous culture. Our findings confirmed the effects of national culture on balance between mathematics achievement and SWB in the sample of 46 countries. Therefore, culture should be a non-negligible factor in the research of mathematics education (Hess et al., 1987; Bishop, 1988; Chen and Uttal, 1988; Stevenson et al., 1993; McInerney et al., 1997; Leung, 2006). Previous studies found large variations between national culture (Hofstede et al., 2010), but the mathematics education worldwide showed the trend of institutional isomorphism (Anderson-Levitt, 2008; George et al., 2019; Kezar and Bernstein-Sierra, 2019). For example, mathematics curriculum around the world has become more uniform (Anderson-Levitt, 2008). Conversely, this study found the culture favoring long-term orientation and restraint could better support students' mathematics achievement and SWB, which indicates the potential benefit of non-western culture, such as Confucian heritage culture. Moreover, borrowing theories may not fit the local culture (e.g., Leung, 2001; Hu, 2019). In this way, mathematics educators should be more confident and humbler to learn from traditions and search for the practice and theory rooted on indigenous cultural values (Leung, 2001).

Third, mathematics educators should focus on the problem of education equity. In this study, students' mathematics achievement and SWB were found to be significantly related to wealth-related variables (family SES and GDP per capita). This result indicates that students from disadvantaged families and regions are more likely to perform worse in mathematics achievement and SWB. In the past, the issue of education equity has received extensive attention in terms of academic achievement. However, the SWB was ignored. This result of this study suggested the importance of research on SWB in providing the full picture of educational equality.

## Conclusion

This study examined the effects of culture on balance between students' mathematics achievement and subjective wellbeing. Results showed the significant effects of cultural dimensions of long-term orientation vs. short-term orientation and indulgence vs. restraint. Students from countries of high long-term orientation and low indulgence culture were more likely to get both high mathematics achievement and high SWB. Besides, wealth-related variables (family SES and GDP per capita) and gender were also found to influence the odds ratio of balance. The findings confirmed the effects of national culture on the balance between mathematics achievement and SWB. Based on the findings, this study discussed the effects of long-term orientation and restraint culture. The results indicate that mathematics educators should consider cultural differences in educational practice and stress the importance and meaning of mathematics learning.

A Few limitations should be noted in this study. First, this study explored the effects of culture at the national level. The results cannot show how culture influences individual learning and subjective feeling. It remains future studies to examine the influence of culture at the individual level. Second, culture is only one of the many influential factors related to the balance between mathematics achievement and SWB. The effects of culture may rely on interactions with other factors, such as national curriculum, classroom teaching, or motivation. Therefore, although this study found the quantitative relationship between culture, mathematics achievement, and SWB, further research is needed to explain the underlying mechanism. Third, since PISA 2018 involved few Muslim and African countries, it may result in bias at the national level.

## Data Availability Statement

The datasets presented in this study can be found in online repositories. The names of the repository/repositories and accession number(s) can be found at: https://www.oecd.org/pisa/test/.

## Author Contributions

JM: conceptualization, methodology, project administration, and funding acquisition. SL: formal analysis, data curation, and visualization. JM and SL: writing-original draft. All authors contributed to the article and approved the submitted version.

## Funding

This research project was supported by Science Foundation of Beijing Language and Culture University (supported by “the Fundamental Research Funds for the Central Universities”, approval number: 22YJ190005).

## Conflict of Interest

The authors declare that the research was conducted in the absence of any commercial or financial relationships that could be construed as a potential conflict of interest.

## Publisher's Note

All claims expressed in this article are solely those of the authors and do not necessarily represent those of their affiliated organizations, or those of the publisher, the editors and the reviewers. Any product that may be evaluated in this article, or claim that may be made by its manufacturer, is not guaranteed or endorsed by the publisher.

## Appendix 1

**Table 1A:** The participating countries and their economic development and cultural values.

**Countries/economies**	**GDP**	**PDI**	**IDV**	**MAS**	**UAI**	**LTO**	**IND**
Argentina	18261.48	49	46	56	86	20	62
Austria	46359.70	11	55	79	70	60	63
Brazil	14347.28	69	38	49	76	44	59
B-S-J-Z (China)	16097.76	80	20	66	30	87	24
Bulgaria	20588.08	70	30	40	85	69	16
Chile	22837.23	63	23	28	86	31	68
Chinese Taipei	47161.39	58	17	45	69	93	49
Colombia	13271.72	67	13	64	80	13	83
Croatia	23331.15	73	33	40	80	58	33
Czech Republic	33179.76	57	58	57	74	70	29
Estonia	30351.51	40	60	30	60	82	16
Finland	41404.71	33	63	26	59	38	57
France	40780.17	68	71	43	86	63	48
Germany	46549.52	35	67	66	65	83	40
Greece	25833.11	60	35	57	112	45	50
Hong Kong	57047.03	68	25	57	29	61	17
Hungary	28358.2	46	80	88	82	58	31
Indonesia	11759.71	78	14	46	48	62	38
Ireland	70747.04	28	70	68	35	24	65
Japan	39316.95	54	46	95	92	88	42
Korea	38467.01	60	18	39	85	100	29
Latvia	26579.18	44	70	9	63	69	13
Lithuania	30742.25	42	60	19	65	82	16
Luxembourg	94521.08	40	60	50	70	64	56
Malaysia	27822.98	104	26	50	36	41	57
Malta	40131.92	56	59	47	96	47	66
Mexico	18319.48	81	30	69	82	24	97
Netherlands	50195.30	38	80	14	53	67	68
Peru	12654.90	64	16	42	87	25	46
Philippines	7946.38	94	32	64	44	27	42
Poland	28439.38	68	60	64	93	38	29
Portugal	28800.89	63	27	31	104	28	33
Romania	23501.42	90	30	42	90	52	20
Russian Federation	25588.94	93	39	36	95	81	20
Serbia	15596.91	86	25	43	92	52	28
Slovak Republic	31221.95	104	52	110	51	77	28
Slovenia	32648.13	71	27	19	88	49	48
Spain	35696.63	57	51	42	86	48	44
Sweden	47674.52	31	71	5	29	53	78
Switzerland	57767.17	34	68	70	58	74	66
Thailand	17313.34	64	20	34	64	32	45
Turkey	24920.01	66	37	45	85	46	49
United Arab Emirates	61510.5	80	38	53	68	23	34
United Kingdom	40644.85	35	89	66	35	51	69
United States	55864.78	40	91	62	46	26	68

*PDI, power distance; IDV, individualism-collectivism; MAS, masculinity-femininity; UAI, uncertainty avoidance; LTO, long-term orientation vs. short-term; IND, indulgence vs. restraint*.
